# Identifying an X-Ray Threshold for Cage Subsidence After Single-Level Minimally Invasive Transforaminal Lumbar Interbody Fusion: A Diagnostic Threshold Study Using Intraoperative CT as the Reference Standard

**DOI:** 10.3390/jcm15124458

**Published:** 2026-06-09

**Authors:** Ahmet Kartal, Gayle R. Salama, Lawrance K. Chung, Noel F. Manalil, Galal A. Elsayed, Roger Härtl

**Affiliations:** 1 Department of Neurological Surgery, Och Spine at NewYork-Presbyterian Hospital, Weill Cornell Medicine, 525 East 68th Street, Box 99, New York, NY 10065, USA; 2 Division of Neuroradiology, Department of Radiology, Weill Cornell Medicine, New York, NY 10065, USA

**Keywords:** cage subsidence, minimally invasive transforaminal lumbar interbody fusion, intraoperative computed tomography, postoperative standing X-ray, diagnostic threshold, ROC analysis

## Abstract

**Background:** Cage subsidence after minimally invasive transforaminal lumbar interbody fusion raises revision risk and costs. Intraoperative computed tomography (CT) provides high-resolution, three-dimensional visualization of the endplate–cage interface and serves as a practical—though itself imperfect—reference standard for early subsidence, but it is not available at all institutions. Plain X-ray is widely available and inexpensive, but lower in resolution. The clinically relevant question is therefore not whether CT and X-ray are equivalent, but rather which X-ray protrusion depth measurement most reliably identifies CT-confirmed subsidence, and whether a positive intraoperative CT meaningfully predicts later radiographic subsidence. **Objective:** Using intraoperative CT as reference, we aimed to (1) determine the optimal X-ray protrusion depth threshold for CT-confirmed early subsidence; (2) test whether intraoperative CT predicts late radiographic subsidence; and (3) examine how early X-ray depth relates to intervertebral disc height (IVDH) and segmental lordosis (SL) loss. **Methods:** In a retrospective single-surgeon cohort (March 2015–July 2023), subsidence was defined as ≥2.0 mm endplate penetration on CT and measured on X-ray by parallax technique. Sensitivity, specificity, accuracy, and Cohen’s κ were calculated. Receiver operating characteristic (ROC) analysis evaluated X-ray depth as a continuous predictor and identified the Youden-optimal cutoff. Intraoperative CT was tested against late radiographic subsidence; no-intercept linear models estimated per-millimeter IVDH and SL loss. **Results:** Of 100 patients, 93 had paired imaging (mean age 66.7 years; body mass index 26.8 kg/m^2^). Subsidence appeared on CT in 16.1% and on X-ray in 15.1%. X-ray showed 80.0% sensitivity, 97.4% specificity, 94.6% accuracy, and κ = 0.80; ROC analysis demonstrated strong discrimination (area under the curve 0.91; 95% confidence interval 0.81–1.00), Youden-optimal cutoff 1.90 mm. Intraoperative CT predicted late subsidence (*n* = 76) with only 45.8% sensitivity and 96.2% specificity; missed cases had penetration depths indistinguishable from non-subsiders. Each 1 mm of early X-ray depth corresponded to 0.45 mm IVDH and 0.37° SL loss. **Conclusions:** An X-ray protrusion depth of 2.0 mm reliably identifies CT-confirmed early subsidence, providing a preliminary diagnostic cutoff for use when CT is unavailable. Intraoperative CT is highly specific but insensitive for late subsidence; meaningful risk stratification will require additional inputs. These hypothesis-generating findings warrant prospective validation.

## 1. Introduction

Cage subsidence, defined as the migration or penetration of an interbody fusion cage into adjacent vertebral endplate(s), remains a persistent clinical challenge in spinal fusion surgery, particularly when expandable cage technologies are employed [1,2,3]. Accurate identification and assessment of subsidence rely on radiographic imaging, primarily plain X-rays and computed tomography (CT) [1,4,5]. Importantly, subsidence warrants focused attention, as it significantly increases the likelihood of revision surgery compared with cases without subsidence, thereby directly influencing patient outcomes and healthcare costs [6,7,8,9].

X-ray imaging, while commonly employed due to its accessibility and cost, has notable limitations. Factors such as variability in patient positioning and insufficient resolution undermine its reliability, particularly when precise millimeter-scale measurements are required to evaluate the severity of subsidence [1,10]. Conversely, CT imaging offers superior spatial resolution and more consistent positioning, potentially enabling more accurate and timely detection of cage subsidence [1,10].

Quantitatively, subsidence is typically measured by assessing the postoperative reduction in intervertebral height. The thresholds for defining significant subsidence vary widely across the literature, reflecting heterogeneous methodologies and criteria; however, most studies commonly reference a threshold of ≥2.0 mm [1,2,11,12]. The 2.0 mm threshold is not arbitrary: it approximates the limit at which endplate penetration begins to exceed the resolution and positioning variability of plain radiography, and in biomechanical and clinical studies it corresponds to the degree of endplate failure associated with measurable loss of disc height, foraminal compromise, and increased reoperation risk, which is why it has become the most frequently adopted cutoff in the subsidence literature. Additionally, subsidence can be evaluated using a grading system (Marchi grading system) based on the percentage of postoperative disc height loss [1,13,14,15], further complicating standardization across studies. Due to variations in definitions and measurement methods, comparing the incidence rates and clinical outcomes of cage subsidence across studies remains challenging.

The etiology of subsidence is multifactorial, with several patient-related risk factors identified, including advanced age, female sex, osteoporosis, high body mass index (BMI), and degenerative disc disease [16,17,18,19,20]. Surgical technique also plays a critical role, with factors such as the surgical approach and potential endplate violation from overly aggressive preparation contributing to subsidence [16,17,18,19,20]. Additionally, the surgeon’s experience significantly affects subsidence rates [21], underscoring the importance of careful torque application during cage expansion. Furthermore, implant-related characteristics, including biomaterial choice (e.g., titanium versus polyetheretherketone), implant dimensions, and positioning, significantly influence the degree of cage subsidence [16,17,18,19,20]. Subsidence is particularly important because it leads to higher rates of pseudoarthrosis and reoperation than in non-subsided cases [6,9]. Surgical complications due to subsidence can increase the economic burden by more than $13,500 USD per case [7,8].

Given the clinical importance of subsidence and the practical reality that CT is not universally available at the point of postoperative care, this retrospective cohort study addresses a diagnostic-threshold question rather than a head-to-head comparison of modalities. Treating intraoperative CT as the reference standard for early cage subsidence, we ask: what protrusion depth measurement on immediate postoperative standing lateral X-ray most reliably identifies CT-confirmed subsidence, and how well does X-ray perform at that threshold? Establishing such a cutoff would provide clinicians with a defensible numerical decision rule for routine radiographic surveillance when CT is unavailable and clarify the conditions under which a positive or negative intraoperative CT should be acted upon.

## 2. Methods

### 2.1. Study Design and Patient Selection

This retrospective cohort study reviewed consecutive patients who underwent minimally invasive one-level transforaminal lumbar interbody fusion (mTLIF) procedures using expandable interbody cages at the Department of Neurological Surgery, Och Spine, New York–Presbyterian Hospital, Weill Cornell Medicine, between March 2015 and July 2023. A total of 100 patients were identified, of whom 93 were included in the study. Patients undergoing revision surgery at the same spinal level for pseudoarthrosis, as well as those with non-degenerative spinal conditions—such as acute traumatic fractures, infectious spondylodiscitis or vertebral osteomyelitis, congenital deformities, inflammatory spondyloarthropathies such as ankylosing spondylitis, or neoplastic lesions—along with patients with poor-quality imaging, were excluded.

All surgeries were performed by a single senior neurosurgeon (R.H.) with extensive experience in minimally invasive spine surgery, specifically mTLIF techniques employing expandable cages.

### 2.2. Imaging Assessment

**Figure 1:**
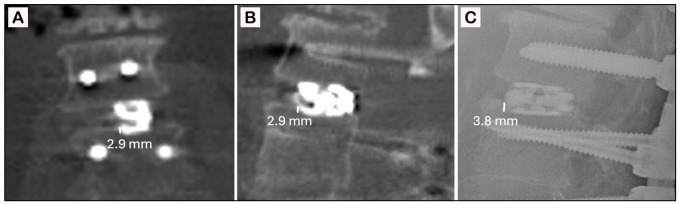
***Modality-Dependent Variation in Interbody Cage Measurement After Lumbar Fusion.*** (**A**) Coronal and (**B**) sagittal reformatted CT images, and (**C**) immediate postoperative lateral radiograph of the same patient after instrumented lumbar interbody fusion. White calipers indicate the endplate–implant measurement used to estimate cage position/subsidence. The measured value differs between CT and X-ray due to modality and projection effects (slice selection, magnification, and patient positioning). ***Abbreviations:*** CT = computed tomography.

Radiographic evaluations were conducted by a single board-certified and experienced neuroradiologist (G.R.S.) and included intraoperative CT scans and immediate postoperative standing lateral X-rays. Cage position was measured with the parallax-phenomenon technique: image stacks were scrolled until the cage’s cranial and caudal margins precisely overlaid the adjacent endplate cortices, eliminating beam-angle distortion and yielding orthogonal, millimeter-accurate measurements. All measurements were performed once by this single reader; repeated or independent dual-reader measurements were not obtained, and formal intra- or inter-rater reliability statistics could therefore not be computed (see Section 4.6). Intraoperative CT scans were performed using the AIRO scanner (Stryker, Portage, MI, USA) according to a standard institutional spine protocol. To minimize patient radiation exposure, the field of view was restricted to the region of interest, and the lowest effective dose was applied. No dedicated metal-artifact-reduction reconstruction algorithm was routinely used. Where streak artifact from instrumentation obscured the endplate–implant interface, the reader selected the least-degraded slice for measurement, and studies of insufficient quality for confident measurement had already been excluded during patient selection. Subsidence was assessed using the predefined 2.0 mm threshold of vertical cage penetration through either vertebral endplate, calculated as the perpendicular distance from the endplate cortex to the apex of cage protrusion (Figure 1A–C). Intervertebral disc height (IVDH) and segmental lordosis (SL) measurements were also obtained from lateral radiographs at the immediate postoperative and follow-up time points to calculate delta (Δ) change values for exploratory analyses.

### 2.3. Statistical Analysis

All statistical analyses were performed using R software (version 4.5.0; R Foundation for Statistical Computing, Vienna, Austria). Continuous data were evaluated for normality using the Shapiro–Wilk test. Normally distributed data were reported as mean ± standard deviation, while non-normally distributed data were summarized as median with interquartile range (IQR). Between-group comparisons for continuous data used either independent two-sided Student’s *t*-tests (parametric) or Mann–Whitney U tests (Wilcoxon rank-sum tests) for non-parametric distributions. Paired categorical outcomes were compared using McNemar’s chi-square test.

Graphical outputs were prepared either in R—with the ggplot2 package—or in GraphPad Prism (version 10; GraphPad Software LLC, Boston, MA, USA).

Univariate logistic regression was conducted to examine potential associations between cage subsidence, evaluated separately for CT and X-ray imaging, and demographic factors (age, BMI, and gender). Results are expressed as odds ratios (OR) with a 95% confidence interval (CI) and associated *p*-values. A two-sided *p*-value < 0.05 was considered statistically significant. Because the number of subsidence events was small, the female-sex association was additionally evaluated with Firth penalized logistic regression as a sensitivity analysis.

Receiver operating characteristic (ROC) analysis was performed in R (pROC package) to evaluate X-ray protrusion depth (mm) as a continuous predictor of CT-confirmed subsidence. The area under the curve (AUC) was calculated with a 95% confidence interval using DeLong’s method. The optimal cutoff was determined by maximizing the Youden index, and sensitivity, specificity, and likelihood ratios (LR+ and LR−) were computed at that threshold. The ROC curve was visualized with ggplot2. Because the AUC and the Youden-optimal cutoff were derived in the same cohort, internal validation was performed to quantify optimism. We used 2000 bootstrap resamples to estimate an optimism-corrected AUC and a 95% bootstrap percentile confidence interval, and we recorded the distribution of the bootstrap-derived Youden cutoff to assess its stability. Leave-one-out cross-validation of a logistic model (CT-confirmed subsidence ~ X-ray depth) was performed as an additional check on discrimination. The optimism-corrected AUC was 0.91 (optimism 0.002), the 95% bootstrap percentile CI was 0.80–0.99, the leave-one-out cross-validated AUC was 0.85, and the bootstrap Youden cutoff had a median of 2.00 mm (95% range 1.0–2.6 mm), with 69% of resamples placing the optimal cutoff between 1.5 and 2.5 mm.

### 2.4. Secondary Analysis for Radiographic Impact of Subsidence Magnitude

Cases with measurable early subsidence on the immediate postoperative X-ray with a protrusion depth ≥ 2.0 mm (i.e., radiographically positive subsidence) and non-missing change values for IVDH Δ and SL Δ (computed as follow-up—immediate postoperative measurement; negative values indicate loss) were then included for secondary analysis. For each outcome, a no-intercept linear regression model was fit (Δ ~ 0 + depth) to force the relationship through the origin (0 mm subsidence assumed 0 change), and the slope was converted to a per-mm loss rate (loss = −slope). All data processing and visualization were performed in R. The readxl, dplyr, and tidyr packages were utilized for data import and wrangling. ggplot2 was used to construct faceted scatter plots with zero-reference and no-intercept regression lines, while gridExtra and grid were used to format the accompanying table detailing predicted losses across common depths (1–5 mm) and per additional 1 mm.

### 2.5. Ethical Considerations

The study was performed in compliance with the Declaration of Helsinki (2013 revision). Approval was granted by the Institutional Review Board of Weill Cornell Medicine (approval number: 24-05027425). Given the retrospective nature of the study, individual informed consent was waived, and patient data were anonymized to protect confidentiality.

## 3. Results

### 3.1. Patient Characteristics and Operated Levels

Among the 93 patients analyzed, the mean age was 66.7 years (95% CI 64.3–69.1), and the mean BMI was 26.8 kg/m^2^ (25.8–27.8); 49/93 (52.7%) were male. The index level was most commonly L4–5 (60/93, 64.5%), followed by L5–S1 (24/93, 25.8%), L3–4 (7/93, 7.5%), and L1–2 and L2–3 (each 1/93, 1.1%). In the CT (+) group (*n* = 15), age and BMI averaged 64.1 years (55.1–73.1) and 26.2 kg/m^2^ (23.7–28.8); 11/15 (73.3%) were female. CT (+) cases occurred at L4–5 in 6/15 (40.0%), L5–S1 in 7/15 (46.7%), L3–4 in 1/15 (6.7%), and L1–2 in 1/15 (6.7%); none were at L2–3. In the X-ray (+) group (*n* = 14), age and BMI averaged 62.8 years (53.8–71.7) and 25.8 kg/m^2^ (23.3–28.3); 10/14 (71.4%) were female. X-ray (+) cases occurred at L4–5 in 5/14 (35.7%), L5–S1 in 6/14 (42.9%), L3–4 in 2/14 (14.3%), and L1–2 in 1/14 (7.1%); none were at L2–3. Table 1 provides a tabular overview of patient demographics and surgical characteristics for this study.

### 3.2. Subsidence Detection Rates and Diagnostic Accuracy

**Figure 2:**
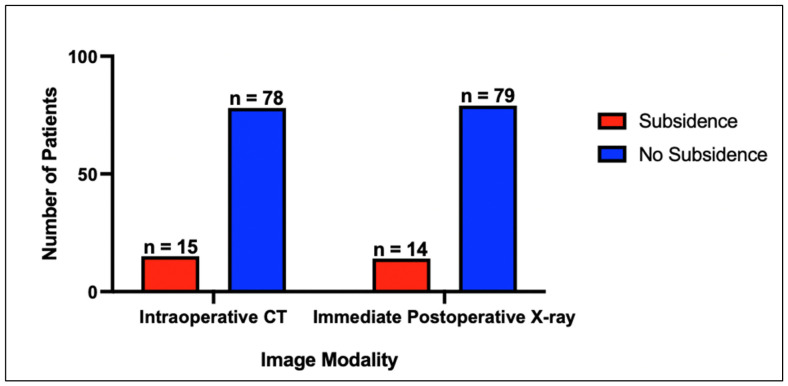
***Classification of Patients at the 2.0 mm Threshold on the Reference-Standard Intraoperative CT and on Immediate Postoperative X-ray.*** Patient counts produced by the prespecified 2.0 mm classification rule applied to each imaging modality. CT serves as the reference standard for early cage subsidence; X-ray protrusion measurements at ≥2.0 mm are the candidate radiographic surrogate. Bars show the number of patients classified as positive (red) or negative (blue) by each modality at the index operation. On reference-standard intraoperative CT, 15/93 (16.1%) patients were classified as having subsidence, and 78/93 as not having subsidence. Applying the same 2.0 mm criterion to immediate postoperative X-ray protrusion depth produced a positive classification in 14/93 (15.1%) and negative in 79/93. Numbers above bars indicate counts. The similarity of marginal positivity rates does not by itself establish modality equivalence; per-patient agreement is reported in Table 2. ***Abbreviations:*** CT = computed tomography.

Of 100 enrolled patients, 93 had paired intraoperative CT and immediate postoperative X-ray available for analysis. Applying the prespecified ≥2.0 mm definition, the reference-standard intraoperative CT classified 16.1% (15/93) of patients as having early cage subsidence. Applying the same 2.0 mm depth criterion to immediate postoperative X-ray protrusion measurements yielded a positive classification in 15.1% (14/93). The two thresholded classifications did not differ on paired testing (McNemar χ^2^ = 0.0 with continuity correction, *p* = 1.00; uncorrected χ^2^ = 0.20, *p* = 0.65; exact two-sided *p* = 1.00; see Figure 2).

### 3.3. Univariable Predictors of Subsidence

On univariable logistic regression for CT-defined subsidence, neither age (OR per year, 0.98; 95% CI, 0.94–1.02; *p* = 0.337) nor BMI (OR per kg/m^2^, 0.97; 0.85–1.09; *p* = 0.592) was associated with subsidence, whereas female sex had higher odds versus male (OR, 3.75; 1.17–14.49; *p* = 0.035). For X-ray-defined subsidence, age (OR per year, 0.97; 0.93–1.02; *p* = 0.174) and BMI (OR per kg/m^2^, 0.94; 0.82–1.07; *p* = 0.389) were not significant; the association with female sex trended toward significance (OR, 3.31; 1.01–12.90; *p* = 0.059). Univariable odds ratios, both overall and for imaging-defined subsidence, are presented in Table 1.

### 3.4. Concordance Between Image Modalities

Using CT as the reference standard and X-ray as the screening test, Table 2 presents the 2 × 2 classification with 12 true positives, 3 false negatives, 2 false positives, and 76 true negatives. X-ray performance was as follows: sensitivity was 80.0% (12 out of 15), specificity was 97.4% (76 out of 78), and accuracy was 94.6% (88 out of 93). The agreement metrics included a positive percent agreement of 85.7% (12 out of 14) and a negative percent agreement of 96.2% (76 out of 79), with Cohen’s κ = 0.80, indicating substantial agreement. Because these estimates are based on only 15 CT-confirmed events, the corresponding sensitivity, specificity, and likelihood-ratio values are necessarily accompanied by wide confidence intervals and should be regarded as preliminary point estimates rather than precise performance figures.

### 3.5. Receiver Operating Characteristic Analysis of X-Ray Protrusion Depth Predicting CT-Confirmed Subsidence

**Figure 3:**
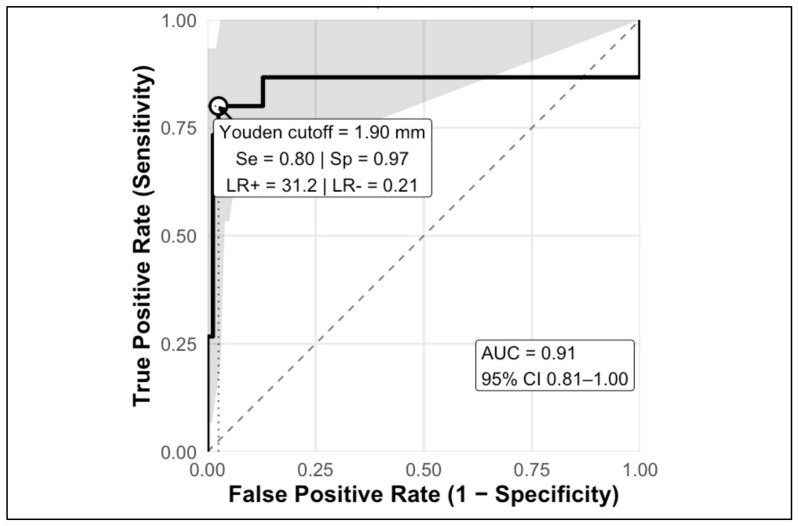
***Receiver Operating Characteristic (ROC) Curve for X-Ray Protrusion Depth Predicting CT-Confirmed Subsidence.*** Receiver operating characteristic analysis evaluating how well X-ray protrusion depth (mm) discriminates patients with CT-confirmed subsidence in a cohort of 93 patients (15 with subsidence). Discrimination was high (AUC = 0.91; 95% CI 0.81–1.00). The Youden index identified an optimal cutoff of 1.90 mm, yielding sensitivity (Se) = 0.80 and specificity (Sp) = 0.97, with likelihood ratios LR+ = 31.2 and LR− = 0.21. The dashed diagonal line represents no-discrimination (chance) performance; the shaded region represents the 95% confidence band around the ROC curve. ***Abbreviations:*** AUC = area under the curve; CI = confidence interval; CT = computed tomography; LR+ = positive likelihood ratio; LR− = negative likelihood ratio; ROC = receiver operating characteristic; Se = sensitivity; Sp = specificity.

ROC analysis showed that X-ray protrusion depth discriminated CT-confirmed subsidence with high accuracy (AUC = 0.91; 95% CI 0.81–1.00; Figure 3). The Youden index identified an optimal cutoff of 1.90 mm, yielding sensitivity = 0.80 and specificity = 0.97 (LR+ = 31.2; LR− = 0.21). Internal validation indicated only modest optimism: the bootstrap optimism-corrected AUC was 0.91 (95% percentile CI 0.80–0.99), the leave-one-out cross-validated AUC was 0.85, and the bootstrap-derived Youden cutoff was stable (median 2.00 mm; 95% range 1.0–2.6 mm), with 69% of resamples placing the optimal cutoff between 1.5 and 2.5 mm. These figures should nonetheless be interpreted in light of the small number of events (*n* = 15) and the absence of external validation.

### 3.6. Patterns of Immediate vs. Delayed (De Novo) Subsidence as Measured on Lateral Radiographs

**Figure 4:**
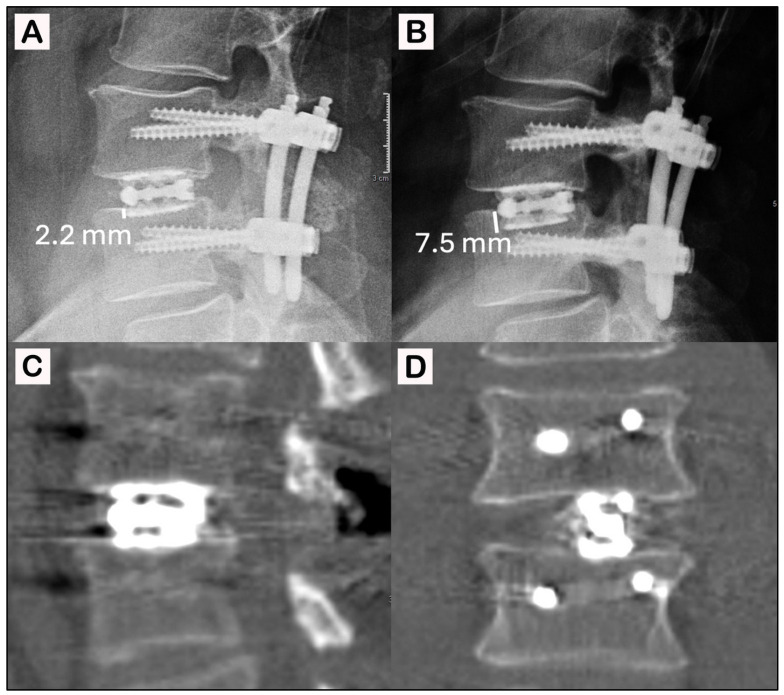
***Progressive Interbody Cage Subsidence Over 1 Year; Early Assessment Limited by Intraoperative CT Streak Artifact.*** (**A**) Immediate postoperative lateral radiograph demonstrates mild subsidence of the interbody spacer. (**B**) Lateral radiograph at approximately 12 months shows marked progression with greater endplate indentation and loss of disc height. (**C**) Sagittal and (**D**) coronal reformats of the intraoperative CT obtained after instrumentation; streak artifact from the hardware obscures the endplate–implant interfaces, making early subsidence difficult to appreciate on intraoperative imaging. Representative patient: 67-year-old male, BMI 30.52 kg/m^2^, operated level L3–4. ***Abbreviations:*** BMI = body mass index; CT = computed tomography; L = lumbar.

The median length of follow-up in our cohort was 12.5 months (IQR 11.2–14.1). Among patients with available follow-up imaging (*n* = 76), subsidence was present in 24/76 (31.6%) and absent in 52/76 (68.4%). On the immediate postoperative radiograph (*n* = 93), subsidence was recorded in 14/93 (15.1%) and absent in 79/93 (84.9%). Among those who ultimately demonstrated subsidence at follow-up (*n* = 24), 12 (50.0%) already had subsidence evident immediately postoperatively, whereas 12 (50.0%) had no immediate subsidence, indicating that exactly half of follow-up cases were de novo. A comparison of patients with versus without late radiographic subsidence (age, sex, BMI, operated level, and early CT/X-ray findings) is provided in Supplementary Table S1. In brief, patients who developed late subsidence did not differ significantly in age (66.4 vs. 67.4 years; *p* = 0.69), BMI (26.4 vs. 26.7 kg/m^2^; *p* = 0.80), or female proportion (54% vs. 48%; *p* = 0.81), but had markedly greater early X-ray protrusion depth (2.0 vs. 0.1 mm; *p* < 0.001) and far higher rates of early CT-confirmed (46% vs. 4%; *p* < 0.001) and early X-ray-confirmed (50% vs. 0%; *p* < 0.001) subsidence; these comparisons are exploratory. These quantitative patterns (Table 3) align with the representative case in Figure 4, which illustrates progressive interbody cage subsidence over one year (Figure 4A,B). Notably, Figure 4C,D highlight intraoperative CT streak artifact at the endplate–implant interface, which can obscure early subsidence and may partly explain why a substantial fraction of patients with later subsidence had negative immediate studies.

### 3.7. Exploratory Impact of Subsidence Magnitude on Disc Height and Segmental Lordosis

**Figure 5:**
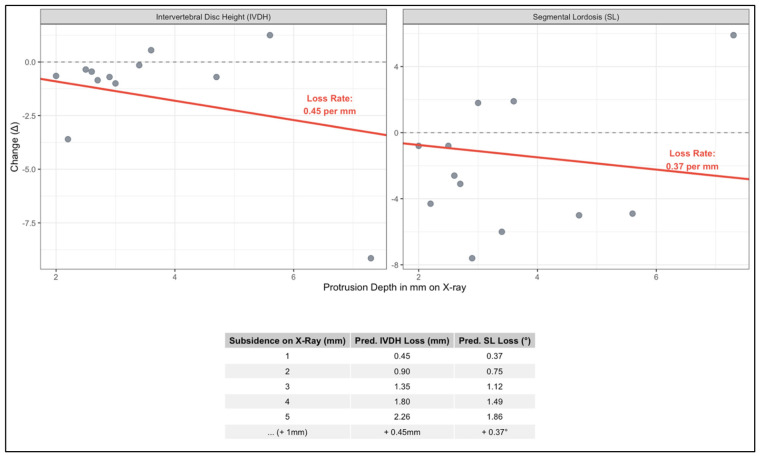
***Impact of Subsidence on Disc Height and Segmental Lordosis.*** Scatter plots showing the relationship between protrusion depth (mm) of early subsidence on the immediate postoperative X-ray and changes (Δ) in intervertebral disc height (IVDH; **left**) and segmental lordosis (SL; **right**). Red lines represent no-intercept linear regression (forced through the origin), yielding mean loss rates of 0.45 mm IVDH and 0.37° SL per additional 1 mm of subsidence. The table summarizes predicted losses for 1–5 mm of subsidence and for each additional 1 mm of subsidence. ***Abbreviations****:* IVDH = intervertebral disc height; Pred. = predicted; SL = segmental lordosis; Δ = change.

In an exploratory secondary analysis, each additional 1 mm of subsidence depth on immediate postoperative X-ray corresponded to an estimated 0.45 mm loss of IVDH and 0.37° loss of SL. Predicted losses across common depths are shown in Figure 5 (e.g., at 2.0 mm: ~0.90 mm IVDH loss and ~0.75° SL loss).

## 4. Discussion

Using intraoperative CT as a practical but imperfect reference standard, the central finding of this study is that an X-ray protrusion depth measurement of approximately 1.90–2.0 mm reliably identifies CT-confirmed early cage subsidence. ROC analysis, treating X-ray depth as a continuous predictor, showed high discrimination for CT-confirmed subsidence (AUC = 0.91; 95% CI 0.81–1.00); the Youden-optimal cutoff was 1.90 mm, which essentially recovers the conventional 2.0 mm radiographic threshold. At the prespecified ≥2.0 mm criterion, X-ray correctly classified most CT-positive cases (sensitivity 80.0%) with very few false positives (specificity 97.4%) and showed substantial agreement with the reference standard (Cohen’s κ = 0.80). Because this cutoff was derived and tested within a single cohort without external validation, it is best regarded as a preliminary, hypothesis-generating diagnostic observation rather than an established clinical decision rule: when CT is unavailable, an X-ray protrusion measurement at or above 2 mm performed well as a surrogate for CT-confirmed subsidence in this cohort, and a measurement well below it corresponded to a low likelihood of CT-confirmed subsidence; these estimates require prospective, external confirmation before clinical adoption.

### 4.1. Technical Comparison of CT Versus X-Ray

CT has several technical advantages compared to X-rays. CT can capture three-dimensional, multiplanar (sagittal, coronal, and axial) images that provide a higher-resolution view of the interbody cage against the endplate, without interference from adjacent structures [22,23], enabling better visualization to determine whether the cage is settling into the cancellous bone. CT can also distinguish between soft tissue, cortical bone, and cancellous bone, offering additional advantages in detecting cage subsidence and associated endplate disruption [24].

An X-ray is a two-dimensional projection image and is limited to anterior–posterior and lateral views. All structures on the same X-ray plane overlap, creating anatomical clutter. As a result, high-intensity structures can obscure subtle low-contrast changes [25]. Many of the X-ray images in this study contained obscured cage-endplate interfaces, making subsidence determination more challenging. The inclusion of hardware, such as pedicle screws or rods, can also obscure the already low-resolution image, further hindering accurate determination of subsidence [26]. Detecting these subtle changes is important for identifying endplate sinking or protrusion caused by the interbody cage, making X-rays inferior to CT scans for detecting subsidence. Beyond resolution, the two modalities differ systematically in geometry, and this is a potential source of measurement bias. Intraoperative CT is acquired supine with the patient anesthetized and provides reformatted orthogonal planes, whereas immediate postoperative radiographs are acquired standing, under physiological axial load, and are subject to magnification and projection effects that depend on patient positioning, source-to-image distance, and beam angulation. These differences can shift millimeter-scale protrusion measurements in either direction and may account for part of the discordance observed between modalities. The parallax-phenomenon technique was used specifically to minimize beam-angle distortion, but it cannot eliminate load- and projection-dependent differences; the resulting agreement statistics should therefore be interpreted as reflecting both true subsidence and modality-specific measurement geometry.

Since intraoperative CT is performed before patient bed repositioning and in more controlled surgical settings, it can detect subsidence immediately after cage expansion. Altered postoperative patient positioning, combined with low-resolution plain radiography, may misrepresent cage stability and delay recognition of complications. The clinical significance of subsidence includes loss of segmental lordosis, pseudoarthrosis, foraminal narrowing, and implant migration [1,27,28]. Early identification of these complications during surgery can prompt immediate intraoperative correction or hardware reinforcement, potentially improving surgical outcomes and reducing the risk of reoperation. However, these downstream clinical outcomes were not assessed in the present study and represent a theoretical rationale rather than a demonstrated benefit.

### 4.2. Intraoperative CT as a Predictor of Late Radiographic Subsidence

A separate question from immediate postoperative subsidence detection is whether intraoperative CT can flag, while the patient is still on the table, those cases destined to develop radiographic subsidence at later follow-up—the time point at which subsidence carries its greatest clinical weight through loss of segmental lordosis, foraminal narrowing, pseudoarthrosis, and the potential need for revision. In this cohort, 24 patients developed late radiographic subsidence. Of these, fewer than half—11 of 24—had already met the prespecified ≥2.0 mm threshold on intraoperative CT, while the remaining 13 showed no intraoperative subsidence despite subsiding later. Among the 52 patients who did not subside on late follow-up, only 2 had crossed the 2.0 mm threshold intraoperatively. Translated into diagnostic-test terms, intraoperative CT at 2.0 mm identified future subsidence with a sensitivity of 45.8% (95% CI 27.9–64.9%) and a specificity of 96.2% (95% CI 87.0–99.0%), a positive likelihood ratio of 11.9, a negative likelihood ratio of 0.56, and a diagnostic odds ratio of 21.2 (Fisher’s exact *p* < 0.001). The clinical interpretation is straightforward: an intraoperative CT showing ≥2.0 mm of cage settling is a strong rule-in signal and should prompt the surgeon to consider on-table corrective measures such as cage height adjustment or repositioning; but an intraoperative CT that shows no subsidence as defined by the 2.0 mm threshold does not reassure, because more than half of the patients who ultimately subsided passed this check unremarked.

We considered whether a less conservative threshold—for example, 1.75 mm, just below the 2.0 mm cutoff—might have captured these missed cases; however, the data do not support this. The 11 measured intraoperative CT subsidence values among future subsiders ranged from 2.7 to 7.5 mm (median 3.3 mm); all already exceeded 2.0 mm, so lowering the threshold to 1.75 mm would not have reclassified any of them. The 13 late-subsiders who would need to be identified using a lower threshold all had intraoperative subsidence of <2.0 mm. For a 1.75 mm cutoff to meaningfully rescue sensitivity, most of these 13 patients would have to fall within the narrow 1.75–2.0 mm band, and any such gain would be offset by an unquantifiable redistribution of the 50 sub-2.0 mm controls across the same band, rendering specificity indeterminate across its full feasible range of 0 to 96%. Moving the threshold in the other direction offers no help either: in this subset, sensitivity falls from 45.8% at 2.0 mm to approximately 33% at 3.0 mm and 8% at 4.0 mm, while specificity is already at the ceiling and cannot meaningfully improve.

The deeper implication is that 13 of 24 future subsiders were genuinely indistinguishable from non-subsiders on intraoperative CT as currently acquired and interpreted—they occupied the same sub-2.0 mm range as the great majority of patients who never went on to subside. No repositioning of the decision threshold on the same measurement scale can separate populations that overlap in this way; the limitation is one of signal, not cutoff. For the spine surgeon, this means that intraoperative CT at its current resolution and reporting convention should be treated as a specific but incomplete safety net—valuable when positive, non-reassuring when negative—and that meaningful improvement in intraoperative risk stratification will require a different input, whether that be quantitative sub-millimeter CT measurement captured prospectively in every case, complementary intraoperative assessment of endplate integrity or bone quality, or preoperative risk stratification by bone mineral density (BMD), rather than further fine-tuning of a binary threshold.

### 4.3. Secondary Risk Factors of Cage Subsidence

Univariable analyses did not identify age or BMI as significant predictors of subsidence on either modality. For CT-confirmed subsidence, female sex was associated with higher odds (OR 3.75, 95% CI 1.17–14.49; *p* = 0.035); the association on radiographs trended similarly (OR 3.31, 95% CI 1.01–12.90; *p* = 0.059). Existing literature indicates that women undergoing lumbar spine surgery may be at increased risk for cage subsidence, potentially due to osteoporotic weakening of the vertebral endplates in contact with the interbody cage [29,30]. Other studies have identified older age as a contributing factor to cage subsidence [30,31]. Advancing age is associated with lower BMD and mechanical integrity, which can reduce the spine’s ability to withstand axial loads [32,33]. However, this association was not observed in our study, likely again due to the limited sample size. BMI was not uniquely associated with cage subsidence, a finding consistent with some reports in the literature [34,35] but inconsistent with others [31,36]. These discrepancies are most likely due to variations in study design, sample size, patient populations, implant types, or definitions of subsidence, all of which can impact the association between BMI and subsidence rates. Importantly, the association between female sex and CT-confirmed subsidence should not be interpreted in isolation. The analysis was univariable; the confidence interval was wide (OR 3.75; 95% CI 1.17–14.49); and no bone-quality data—BMD, dual-energy X-ray absorptiometry findings, CT Hounsfield units, or osteoporosis treatment history—were available. Because female sex in this age range is a strong proxy for lower BMD, this association is most plausibly confounded by unmeasured bone quality rather than representing an independent effect of sex, and it should be regarded as hypothesis-generating. In a sensitivity analysis using Firth penalized logistic regression, which is appropriate when the number of events is small, this association attenuated but remained directionally consistent (penalized OR 3.47, 95% CI 1.13–12.49, versus the standard OR 3.75), confirming that the point estimate is unstable.

### 4.4. Comparative Analysis of CT and X-Ray Imaging in the Literature

The X-ray subsidence rate of 15.1% falls within the range (4.1% to 31.8%) reported in previous follow-up imaging studies [1,18,37]. The CT-scan subsidence rate of 16.1% falls below the reported range of 21.4% to 50.6% (Supplementary Table S2). In our cohort, the proportion classified as subsided at the prespecified ≥2.0 mm criterion was 16.1% on the reference-standard intraoperative CT and 15.1% on immediate postoperative X-ray. The marginal proportions are similar, but this is not the central finding of our analysis: similar positivity rates do not establish modality equivalence; per-patient agreement, rather than overall rate, is the meaningful comparison. The clinically relevant question that this study was designed to answer is what X-ray cutoff predicts CT-confirmed subsidence, not whether X-ray and CT yield the same overall positivity rate. Our ROC analysis (AUC 0.91) and the 1.90 mm Youden-optimal cutoff support continued use of plain radiographs at the conventional 2.0 mm threshold for routine postoperative surveillance when CT is unavailable.

### 4.5. Other Factors to Address in Postoperative Spine Imaging

It is important to note that CT modalities and plain radiographs in postoperative spine imaging assessment may differ in their diagnostic performance. Park et al. found that CT overestimates fusion rates compared to X-rays following spinal fusion surgery [38]. Radiographs can capture dynamic instability through flexion-extension views, whereas CT scans can only evaluate static features [38]. CT is useful for detecting hardware loosening or malunion, but it is less sensitive for identifying true arthrodesis [39]. Thus, the optimal evaluation of postoperative spine fusions may require a combination of dynamic plain radiographs and high-resolution static CT imaging.

### 4.6. Limitations

This study has several limitations inherent to its retrospective cohort design. First, all radiographic measurements were performed by a single board-certified neuroradiologist, and neither independent dual-reader measurements nor repeated measurements by the same reader were obtained. Consequently, formal inter-rater and intra-rater reliability for the millimeter-scale CT and X-ray measurements (e.g., the intraclass correlation coefficient or Cohen’s kappa) could not be quantified. Because the diagnostic argument rests on the reproducibility of millimeter-scale measurements, this is an important limitation; prospective work should incorporate independent dual-reader assessment with formal reliability statistics, and the present single-reader estimates may understate measurement variability. Second, the lack of a universally accepted gold standard for detecting cage subsidence may impact diagnostic accuracy; to address this, we compared our observed subsidence rates with published benchmarks. In particular, intraoperative CT is itself an imperfect reference rather than a true gold standard: streak artifact from instrumentation can obscure the endplate–implant interface (Figure 4C,D), and no dedicated metal-artifact-reduction protocol was routinely applied. If the reference standard is systematically insensitive to early subsidence because of metal artifact, the X-ray sensitivity and specificity estimated against it may be biased in ways that cannot be fully corrected post hoc; the analysis is therefore best understood as an evaluation of agreement between two early imaging measurements rather than validation against an independent truth standard. Third, the relatively small sample size and limited number of subsidence events reduce statistical power, leading to wide confidence intervals and potentially limiting the ability to detect clinically relevant effects, especially in X-ray-based analyses. Additionally, no prior power analysis was conducted; thus, this work should be viewed as exploratory and hypothesis-generating. Furthermore, logistic regression results should be interpreted cautiously, given the small number of subsidence events, particularly in X-ray imaging. Fourth, our analysis relied solely on immediate postoperative imaging, excluding delayed subsidence events that might occur later in the postoperative period. Follow-up imaging, when available, consisted of standing lateral radiographs, and the median follow-up of 12.5 months is relatively short for capturing delayed subsidence and its long-term clinical consequences; longer-term studies are needed. In addition, follow-up imaging was available in only 76 of 93 patients, so the late-subsidence analyses are restricted to that subset and may be subject to attrition bias. Fifth, all procedures were performed by a single experienced surgeon at a single center; while this improves procedural consistency, it limits the external validity and generalizability of the findings to other surgeons, techniques, and institutions. Lastly, as an observational study, it faces inherent limitations, including selection bias, unmeasured confounding factors (e.g., bone density, variability in surgical technique, and implant size), and measurement errors in radiographic assessments. Moreover, the ROC analysis (Figure 3) and the 1.90 mm Youden cutoff were derived from the same cohort (15 CT-positive events). Although bootstrap internal validation and leave-one-out cross-validation indicated only modest optimism and a stable cutoff (see Section 3), no external validation was performed; given the small number of events, the estimated AUC, confidence band, and threshold performance may still be optimistic and require validation on an independent dataset. Additionally, the per-mm estimates linking early subsidence depth to intervertebral disc height and segmental lordosis change (Figure 5) were based on a limited subset of cases with complete measurements and modeled as a linear, no-intercept relationship; these assumptions, along with the small sample size, may limit generalizability and warrant validation.

### 4.7. Future Research Directions

Intraoperative CT requires advanced imaging infrastructure that may not be available in community hospitals or resource-limited settings. Cost-effectiveness analyses could assess whether the benefits of intraoperative CT outweigh its higher upfront costs compared to immediate postoperative X-rays. Future research should examine the relevance of subsidence detection using intraoperative CT in association with outcomes such as fusion rates, reoperation rates, and symptom alleviation. Additionally, studies with larger cohort sizes are needed to better conclude associations between demographic risk factors and cage subsidence. As a preliminary exploration of clinical relevance, we examined the association between subsidence and revision surgery in this cohort. The overall reoperation rate was 12% (12/100). Because reoperation status was available for the entire operative cohort, these exploratory associations were assessed in all 100 patients rather than the 93-patient paired-imaging subset; consequently the denominators sum to 100 and include one CT-confirmed case that lacked an analyzable paired radiograph (16 here versus the 15 in the paired-imaging analyses), and the 76 patients in the late-radiographic-subsidence comparison denote the remainder of the 100-patient cohort rather than the 76 patients who had follow-up imaging. Reoperation was not significantly associated with early CT-confirmed subsidence (1/16 [6%] vs. 11/84 [13%]; Fisher exact *p* = 0.69), early X-ray-confirmed subsidence (1/14 [7%] vs. 11/86 [13%]; *p* = 1.00), or late radiographic subsidence (4/24 [17%] vs. 8/76 [11%]; *p* = 0.48). These null associations should be interpreted cautiously: the cohort was underpowered for revision as an outcome (only 12 events), most revisions in spine practice are driven by factors other than radiographic subsidence, and patient-reported outcomes (VAS and ODI) were available in only a minority of patients (approximately one-third) and were therefore too sparse to support a reliable subsidence-stratified analysis. Adequately powered prospective studies linking quantitative subsidence to revision, pseudoarthrosis, and patient-reported outcomes remain an important next step.

## 5. Conclusions

Using intraoperative CT as a practical but imperfect reference standard, this study supports an X-ray protrusion depth measurement of approximately 1.90–2.0 mm as a reliable indicator of CT-confirmed early cage subsidence (AUC = 0.91; 95% CI 0.81–1.00; Youden-optimal cutoff 1.90 mm). The principal contribution of this work is therefore not a head-to-head ranking of CT against X-ray, but a preliminary, hypothesis-generating diagnostic cutoff: when CT is unavailable, an X-ray protrusion measurement at or above the conventional 2.0 mm threshold performed well as a surrogate for CT-confirmed subsidence in this cohort. Because the threshold was developed and tested within the same cohort without external validation, it should be considered preliminary and requires prospective, multicenter confirmation before routine clinical adoption. At this threshold, X-ray sensitivity was 80.0% (12/15) and specificity was 97.4% (76/78), with substantial agreement against CT (Cohen’s κ = 0.80). In the exploratory secondary analysis, each additional 1 mm of early X-ray protrusion depth corresponded to an estimated 0.45 mm loss of intervertebral disc height and a 0.37° loss of segmental lordosis; these associations were exploratory, were based on a small subset of cases, and do not by themselves establish clinically meaningful functional consequences. A separate question—whether intraoperative CT, while the patient is still on the table, predicts subsidence at later follow-up—was answered less favorably: intraoperative CT at the same 2.0 mm threshold was highly specific (96.2%) but only 45.8% sensitive for late radiographic subsidence, and the patients missed intraoperatively were genuinely indistinguishable from non-subsiders on the same measurement scale. A positive intraoperative CT is therefore an actionable rule-in finding, but a negative intraoperative CT does not rule out later subsidence, and meaningful improvement in intraoperative risk stratification will require additional inputs rather than further tuning of a binary depth threshold. Female sex was associated with CT-confirmed subsidence; age and BMI were not significant predictors in this cohort. Taken together, these findings characterize the diagnostic performance of an X-ray protrusion depth cutoff within this single-center cohort and clarify the present limits of intraoperative CT; broader clinical recommendations will require external validation in larger, prospective, multicenter studies.

## Figures and Tables

**Table 1:** ***Patient Demographics, Operated Levels, and Univariable Odds Ratios, Overall and Stratified by Subsidence on Imaging.***

Analysis of Patient Demographics & Odds Ratios by Imaging Modality			
Age/BMI: mean (95% CI); Gender: *n* (%) | OR (95% CI), *p*-value for each predictor			
	**Imaging Modality**		
**Variable**	**Overall (*n* = 93)**	**CT (+) (*n* = 15)**	**X-ray (+) (*n* = 14)**
Age, years	66.75 (64.33–69.18)	64.07 (55.06–73.08)	62.76 (53.84–71.68)
BMI, kg/m^2^	26.81 (25.84–27.79)	26.21 (23.67–28.76)	25.81 (23.28–28.33)
Gender (Male)	49 (52.7%)	4 (26.7%)	4 (28.6%)
Gender (Female)	44 (47.3%)	11 (73.3%)	10 (71.4%)
**Operated level (n, %)**			
L1–2	1 (1.1%)	1 (6.7%)	1 (7.1%)
L2–3	1 (1.1%)	0 (0.0%)	0 (0.0%)
L3–4	7 (7.5%)	1 (6.7%)	2 (14.3%)
L4–5	60 (64.5%)	6 (40.0%)	5 (35.7%)
L5–S1	24 (25.8%)	7 (46.7%)	6 (42.9%)
**Odds Ratios (95% CI), *p*-value**			
Age (per year)		0.98 (0.94–1.02), *p* = 0.337	0.97 (0.93–1.02), *p* = 0.174
BMI (per unit)		0.97 (0.85–1.09), *p* = 0.592	0.94 (0.82–1.07), *p* = 0.389
Gender (Female vs. Male)		3.75 (1.17–14.49), *p* = 0.035	3.31 (1.01–12.90), *p* = 0.059

Continuous variables (Age, BMI) are reported as mean (95% CI); categorical variables (Gender, Operated level) as *n* (%). CT (+) and X-ray (+) denote the presence of subsidence on CT and on standing radiographs, respectively (subgroup denominators *n* = 15 and *n* = 14). Odds ratios (ORs) are from separate univariable logistic regressions for each predictor, with the imaging outcome as the outcome; values are shown as OR (95% CI) and the corresponding Wald *p*-value. Age is modeled per year, and BMI per kg/m^2^; gender is coded Female vs. Male (Male as the reference). The operated level reflects the index level treated. Two-sided tests, α = 0.05. ***Abbreviations***: BMI = body mass index; CI = confidence interval; CT = computed tomography; *n* = number of patients; OR = odds ratio.

**Table 2:** ***Diagnostic Concordance Between Intraoperative CT and Immediate Postoperative X-ray.***

Diagnostic Concordance Between Intraoperative CT and Immediate Postoperative X-Ray			
	Immediate Postoperative X-Ray (+)	Immediate Postoperative X-Ray (−)	Total
**Intraoperative CT (+)**	12	3	15
**Intraoperative CT (−)**	2	76	78
**Total**	14	79	93

Applying the 2.0 mm criterion to X-ray protrusion depth produced a positive classification in 15.1% of cases (14/93). Of the 15 CT-confirmed events, 12 (80%) were correctly identified by X-ray (sensitivity 80%; specificity 97.4%; Cohen’s κ = 0.80, substantial agreement). McNemar’s test showed no significant asymmetry between the two thresholded classifications (χ^2^ = 0.0 with continuity correction, *p* = 1.00; uncorrected χ^2^ = 0.20, *p* = 0.65; exact two-sided *p* = 1.00). ***Abbreviations***: CT = computed tomography; κ = Cohen’s kappa; χ^2^ = chi-square statistic.

**Table 3:** ***Immediate vs. Follow-Up Subsidence as Measured on Lateral Radiographs.***

Immediate vs. Follow-Up Subsidence			
	Immediate Postoperative Subsidence (+)	Immediate Postoperative Subsidence (−)	Total
**Follow-up Subsidence (+)**	12 (50.0%)	12 (50.0%)	24
**Follow-up Subsidence (−)**	0 (0%)	52 (100%)	52
**Total**	12	64	76

Matrix displaying the relationship between subsidence on the immediate postoperative X-ray and on follow-up imaging, restricted to the 76 patients with a known follow-up result. Cells are shown as *n* (row %). Of the 24 patients with subsidence at follow-up [Follow-up Subsidence (+)], 12 (50.0%) already had subsidence on the immediate postoperative X-ray [Immediate Postoperative Subsidence (+)], whereas 12 (50.0%) did not, indicating new/de novo subsidence by follow-up. Among the 52 patients without follow-up subsidence [Follow-up Subsidence (−)], none (0%) had immediate postoperative subsidence; all 52 (100%) did not. Thus, every patient who did not subside at follow-up also had a negative immediate postoperative X-ray. The Total row gives the column totals for this follow-up cohort: immediate postoperative subsidence was present in 12 and absent in 64 of the 76 patients. For context across the full paired cohort (*n* = 93), immediate postoperative subsidence was present in 14 and absent in 79. ***Abbreviations***: (+) = subsidence present; (−) = subsidence absent; *n* = number of patients.

## Data Availability

De-identified data supporting this study’s findings and the R analysis scripts will be made available upon reasonable request, subject to institutional privacy and data use policies.

## Supplementary Materials

The following supporting information can be downloaded at: https://www.mdpi.com/article/10.3390/jcm15124458/s1. Table S1: Comparison of Patients with Versus Without Late Radiographic Subsidence; Table S2: Cage Subsidence Rates Following TLIF Surgery by Imaging Modality, Measurement Thresholds, and Cage Designs.
