# Supplementary material for: Identifying an X-Ray Threshold for Cage Subsidence After Single-Level Minimally Invasive Transforaminal Lumbar Interbody Fusion: A Diagnostic Threshold Study Using Intraoperative CT as the Reference Standard
Source: J Clin Med. 2026 Jun 9;15(12):4458. doi: 10.3390/jcm15124458 (PMC13302173; doi:10.3390/jcm15124458)
Supplement: Supplementary file 1 [file jcm-15-04458-s001.zip › jcm-4331698-supplementary.pdf]

Journal of Clinical Medicine

MDPI

Table S1 (Supplementary). Comparison of Patients With Versus Without Late Radiographic Subsidence.

Characteristic	Late subsidence (*n* = 24)	No late subsidence (*n* = 52)	*p*-value
Age (years), mean ± SD	66.4 ± 9.7	67.4 ± 10.4	0.69
BMI (kg/m^2), mean ± SD	26.4 ± 3.9	26.7 ± 5.1	0.80
Female sex, n (%)	13 (54%)	25 (48%)	0.81
Operated level, n (%)			0.72
L2–3	0 (0%)	1 (2%)	
L3–4	2 (8%)	4 (8%)	
L4–5	14 (58%)	35 (67%)	
L5–S1	8 (33%)	12 (23%)	
Early X-ray depth (mm), mean ± SD	2.0 ± 2.0	0.1 ± 0.4	<0.001
Early CT-confirmed subsidence, n (%)	11 (46%)	2 (4%)	<0.001
Early X-ray-confirmed subsidence, n (%)	12 (50%)	0 (0%)	<0.001

Continuous variables compared with the Mann–Whitney U test; categorical variables with Fisher's exact test (operated level by Fisher-Freeman-Halton/χ^2). Among 93 paired patients, 76 had follow-up imaging. All comparisons are exploratory and unadjusted.

Abbreviations: BMI = body mass index; CT = computed tomography; n = number of patients; SD = standard deviation.

Table S2 (Supplementary). Reported Cage Subsidence Rates in TLIF Studies by Imaging Modality, Measurement Threshold, and Cage Design.

Imaging Modality	Study (Year)	Surgical Technique	Subsidence Rate	Subsidence Criteria
**X-ray**	Park et al. (2019)	TLIF	36/881 (4.1%)	>2 mm
	Zhao et al. (2020)	TLIF	Low grade: 9/76 (11.8%)<br>High grade: 6/76 (7.9%)	>25% (low)<br>>50% (high)
	Mun et al. (2019)	TLIF	25.3%	>2 mm
	Lin et al. (2018)	TLIF	6/20 (30.0%)	>2 mm
	Kim et al. (2013)	TLIF	10/122 (8.2%)<br>8/122 (6.6%)	>2 mm and >4 mm
	Choi et al. (2018) (Banana cages)	TLIF	14/44 (31.8%)	>2 mm
	Choi et al. (2018) (Straight cages)	TLIF	7/40 (17.5%)	>2 mm
**CT**	Pereira et al. (2018)	TLIF	25/117 (21.4%)	>3 mm
	Lin et al. (2017)	TLIF	14/30 (46.7%)	>2 mm
	Lee et al. (2017)	TLIF	8/21 (38.1%)	Not specified
	Nemoto et al. (2014)	TLIF	8/23 (35%) Titanium<br>7/28 (28%) PEEK	>2 mm
	Pisano et al. (2020)	TLIF	45/89 (50.6%)	>2 mm

Adapted from Parisien et al., 2022,[1] with additional TLIF studies and selective extraction of TLIF-specific cohorts from comparative studies. Included papers comprise a mix of confirmed MIS-TLIF studies, open/mini-open TLIF studies, and TLIF studies whose accessible bibliographic record does not specify a minimally invasive approach.

***Abbreviations**:* CT = computed tomography; PEEK = polyetheretherketone; TLIF = transforaminal lumbar interbody fusion.

https://doi.org/10.3390/jcm15124458
